# Radioprotective Effects of Dermatan Sulfate in a Preclinical Model of Oral Mucositis—Targeting Inflammation, Hypoxia and Junction Proteins without Stimulating Proliferation

**DOI:** 10.3390/ijms19061684

**Published:** 2018-06-06

**Authors:** Sylvia Gruber, Marlene Arnold, Nilsu Cini, Victoria Gernedl, Sabine Hetzendorfer, Lisa-Marie Kowald, Peter Kuess, Julia Mayer, Susanne Morava, Stephanie Pfaffinger, Andreas Rohorzka, Wolfgang Dörr

**Affiliations:** 1 Christian Doppler Laboratory for Medical Radiation Research for Radiation Oncology, Medical University/AKH Vienna, 1090 Vienna, Austria; peter.kuess@meduniwien.ac.at; 2 Department of Radiation Oncology, Applied and Translational Radiobiology, Medical University Vienna, 1090 Vienna, Austria; marlene.arnold@meduniwien.ac.at (M.A.); victoria.gernedl@meduniwien.ac.at (V.G.); sabine.hetzendorfer@meduniwien.ac.at (S.H.); lisa_kowald@hotmail.com (L.-M.K.); julia_mayer92@gmx.at (J.M.); susanne.morava@meduniwien.ac.at (S.M.); stephanie.pfafffinger@meduniwien.ac.at (S.P.); andreasrohorzka@gmx.at (A.R.); wolfgang.doerr@meduniwien.ac.at (W.D.); 3 Department of Radiation Oncology, Kartal Dr. Lutfi Kırdar Training and Research Hospital, Health Science University, 34722 Istanbul, Turkey; nilsu.cini@gmail.com

**Keywords:** oral mucositis, mouse model, dermatan sulfate, fractionation, inflammation, hypoxia, cellular junctions

## Abstract

Oral mucositis is the most frequently occurring early side effect of head-and-neck cancer radiotherapy. Systemic dermatan sulfate (DS) treatment revealed a significant radioprotective potential in a preclinical model of oral mucositis. This study was initiated to elucidate the mechanistic effects of DS in the same model. Irradiation comprised daily fractionated irradiation (5 × 3 Gy/week) over two weeks, either alone (IR) or in combination with daily dermatan sulfate treatment of 4 mg/kg (IR + DS). Groups of mice (*n* = 5) were sacrificed every second day over the course of 14 days in both experimental arms, their tongues excised and evaluated. The response to irradiation with and without DS was analyzed on a morphological (cell numbers, epithelial thickness) as well as on a functional (proliferation and expression of inflammation, hypoxia and epithelial junction markers) level. The mucoprotective activity of DS can be attributed to a combination of various effects, comprising increased expression of epithelial junctions, reduced inflammation and reduced hypoxia. No DS-mediated effect on proliferation was observed. DS demonstrated a significant mucositis-ameliorating activity and could provide a promising strategy for mucositis treatment, based on targeting specific, radiation-induced, mucositis-associated signaling without stimulating proliferation.

## 1. Introduction

Despite constant technological advances in conformal radiotherapy, normal tissue effects of radiotherapy cannot be avoided and remain a prominent clinical factor. During radiotherapy of the head-and-neck region, oral mucositis is the most frequently occurring early normal tissue complication. The mucosal radiation response, characterized by painful ulcerative lesions throughout the oral cavity, occurs in the majority of patients and is associated with numerous debilitating effects [1]. The patient´s quality of life is significantly reduced due to mucositis-associated pain, reduced speaking- and swallowing capabilities; the latter can necessitate parenteral nutrition. Furthermore, oral mucositis patients are at risk of local and systemic infections due to the mucosal barrier breakdown [2,3]. Up to date the treatment of oral mucositis solely relies on preventive strategies such as improvement of oral hygiene and mitigation of pain [4,5]. The only biology-based treatment option for oral mucositis so far is the recombinant human keratinocyte growth factor (KGF) palifermin. Administration is recommended only for patients undergoing myoablative irradiation regimes [6]. Due to palifermin being a mitogen for normal and some malignant epithelial cells, including those of the head-and-neck, tumor protection is a major concern. Despite studies indicating no proliferative stimulation of head-and-neck squamous cell carcinoma cells by exogenous KGF [7,8], the concern of tumor protection and the significant costs of palifermin so far hindered its recommendation for head-and-neck cancer patients [9,10]. Ideally, any mitigative approach to oral mucositis is not based on growth factor activity, but rather on other mucositis-relevant biological mechanisms. With recent advances in the field of molecular radiobiology, insights in the molecular events of mucositis development have been gained. The paradigm shifts from mucositis being based solely on radiation-induced inhibition of proliferation and cell death, with subsequent mucosal hypoplasia and ulceration to a multifactorial process [11].

Recently, we described the mucoprotective effect of systemic dermatan sulfate (DS) treatment in a preclinical model of oral mucositis [12]. DS belongs to the family of glycosaminoglycans (GAGs), complex polysaccharides composed of alternating units of uronic acid and hexosamines, with varying amounts of sulfate groups in different positions. Although the polysaccharide backbones of GAGs are linear and relatively simple, sulfation and epimerization give some GAGS, including DS, considerable structural variability, leading to a variety of biological activities that could form the basis of the observed mucoprotective effects [13]. DS exists either as a solute or linked to a protein core, constituting a proteoglycan. Both interact with a multitude of proteins, inducing profound effects on various physiological processes, such as inflammation, coagulation, extracellular matrix assembly and wound healing. DS has been shown to be released in high concentrations during wound repair, promoting cell proliferation and migration via basic fibroblast growth factor (FGF-2) and keratinocyte growth factor KGF [14]. Although being a promising candidate for multiple indications, clinical application of DS are scarce and so far only constitute wound healing and anti-coagulatory strategies. Recently, an in-vitro study demonstrated stimulation of wound repair for a DS containing glycosaminoglycan mixture, which was based on stimulated cell migration and reduced inflammation [15]. Furthermore, DS was evaluated as an anti-coagulation strategy for patients with bleeding disorders, yielding a predictable dose response and overall clinical safety [16,17]. Both, inflammation and hypoxia, as well as chemokine and cytokine release have recently been implicated in mucositis development and therefore present targeted treatment options [18,19,20]. Based these properties, DS was initially chosen to be tested as a mucositis mitigation strategy.

The present study was initiated to elucidate the mechanistic effects of DS in the preclinical mucositis mouse model with emphasis on mucositis-associated protein expression and signaling changes. Epithelial morphology, proliferation and signaling were assessed over the course of two weeks of fractionated irradiation with and without systemic DS administration. Interleukin-1β (IL-1β) is a potent cytokine and regulator of the innate immune response, released early during mucositis development [20,21], stimulating its own synthesis and nuclear factor kappa-light-chain-enhancer of activated B cells (NF-κB) activation via feedback loops. NF-κB is an evolutionary conserved signaling module, inducible by either the canonical (classical) or the alternative pathway. Canonical NF-κB signaling is activated by pro-inflammatory stimuli and appears to be crucial for mucositis development [22]. The five members of the mammalian NF-κB family constitute p50, p52, RelA (p65), RelB and c-Rel, forming hetero- or homodimers, bound to inhibitory proteins in the cytoplasm [23]. Upon stimulation, the NF-κB inhibitory complex is degraded and NF-κB dimers translocate to the nucleus, where they bind target gene promoter or enhancer sequences and activate transcription. Predominant complexes of the canonical pathway are p50:p65 and p50:c-Rel [24]. Hence, p50, being a component of both predominant dimers, was chosen to be evaluated as a NF-κB marker in this study. 

Early hypoxia during fractionated irradiation was recently described to be associated with mucositis development [19,25]. Hypoxia inducible factor-1α (HIF-1α) and glucose transporter-1 (GLUT1) were analyzed as markers of hypoxia. An intricate interaction between hypoxia and inflammatory changes has been proposed for mucositis development [26,27]. Mucosal integrity is maintained via tight and adherens junction complexes. Tight junctions, composed of claudin and occludin proteins, regulate epithelial permeability and integrity. In adherens junctions, the transmembrane proteins e-cadherin and β-catenin together with α-catenin form complexes that bind the actin cytoskeleton force-dependently. This conditions the polarized epithelial phenotype, cell-cell and cell-matrix adhesion [28,29]. To evaluate the effect of DS on epithelial integrity, tight as well as adherens junction markers, claudin-1, occludin, e-cadherin and β-catenin were assessed in the present study.

## 2. Results

### 2.1. Epithelial Morphology and Proliferation

**Figure 1:**
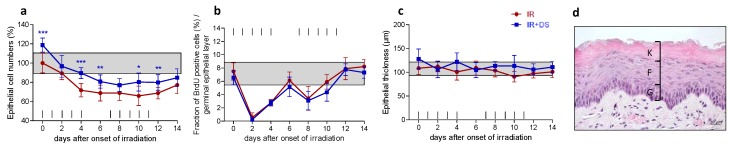
Effect of DS on irradiation-induced changes of epithelial morphology and proliferation. Epithelial cell numbers (**a**); proliferation (**b**) and epithelial thickness (**c**) were assessed over the course of 14 days of fractionation with a daily dose of 3 Gy without and with additional systemic DS treatment. The epithelium is composed of a germinal layer (G), a functional layer (F) and a fully keratinized superficial layer (K). Cell number and proliferation were assessed in the germinal and the functional layer. To evaluate the epithelial thickness, germinal, functional and keratinized layer dimensions were combined (**d**). Data points represent the mean of 5 animals, error bars indicate ±1 standard deviation (SD). The shaded areas illustrate the mean (±1 SD) from 5 control animals. The fractionation protocol is indicated on top of the abscissae. * *p* ≤ 0.05, ** *p* ≤ 0.01, *** *p* ≤ 0.001, Scale bar = 20 µm.

Epithelial cell numbers of untreated and unirradiated control specimen were normalized to 100%. Irradiation alone gradually reduced cell numbers until day 6. The onset of repopulation in the beginning of the second treatment week resulted in a stabilization of cell numbers throughout the rest of the treatment time (Figure 1a). Irradiation rapidly abolished proliferation, evident by a reduction of Bromodeoxyuridine BrdU-positive cell from 8% in control specimen to 0.5% on day 2. The onset of repopulation led to a recovery of normal proliferation from the second treatment week onwards (Figure 1b). Epithelial thickness, composed of the germinal-, the functional-, and the fully keratinized superficial layer (Figure 1d), was not affected by irradiation (Figure 1c). Additional DS application had no effect on epithelial BrdU incorporation but, however, increased epithelial cell numbers throughout the study period. Significantly higher cell numbers were found on day 0 (*p* ≤ 0.001) after DS pretreatment from day-3 onwards, on day 4 (*p* ≤ 0.001), day 6 (*p* = 0.005), day 10 (*p* = 0.018) and day 12 (*p* = 0.007). No significant effects of DS on epithelial thickness were observed throughout the study period.

### 2.2. Epithelial Cell Junctions

#### 2.2.1. Adherens Junctions—β-Catenin and e-Cadherin

**Figure 2:**
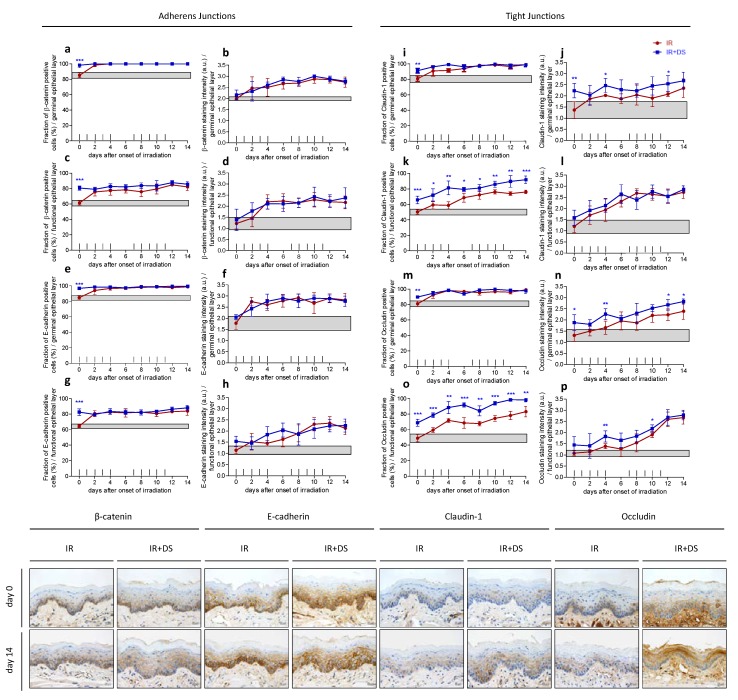
Effect of DS on irradiation-induced epithelial junctions. The relative number of adherens junction marker β-catenin and e-cadherin- (**a**–**h**) or tight junction marker claudin-1 and occludin positive epithelial cells (**i**–**p**) and their respective staining intensity were analyzed, in the germinal and functional compartments, respectively. Data points represent the mean of 5 animals, error bars indicate ±1 SD. The shaded areas illustrate the mean (±1 SD) from 5 control animals. The fractionation protocol is indicated on top of the abscissae. Histophotographs illustrate representative junction marker staining in on day 0 and after irradiation without and with DS on day 14 of the study period. * *p* ≤ 0.05, ** *p* ≤ 0.01, *** *p* ≤ 0.001, Scale bar = 20 µm.

Control specimen displayed approximately 85% of germinal and 60% of functional epithelial cells positive for β-catenin and e-cadherin. With the onset of fractionation, the expression of both adherens junction marker proteins increased to roughly 100% in the germinal tissue compartment (Figure 2a,e) and to levels between 75% and 83% in the functional layer (Figure 2c,g). β-catenin staining intensity increased from 2 arbitrary units (a.u.) on day 0 to 2.7 a.u. (days 10 to 14) in the germinal compartment (Figure 2b) and from 1.2 a.u. (day 0) to 2.3 a.u. (day 10) in the functional layer (Figure 2d). E-cadherin staining intensity increased from 1.8 a.u. (day 0) to 2.8 a.u. (day 12) in the germinal layer (Figure 2f) and from 1.1 a.u. (day 0) to 2.4 a.u. on day 12 in the functional layer (Figure 2h).

Additional DS treatment increased the expression of β-catenin to 97% (*p* ≤ 0.001) and e-cadherin to 98% (*p* ≤ 0.001) in the germinal epithelial layer during the pretreatment period starting on day-3 before the first fraction. Afterwards, expression levels of about 100% were observed with no difference to irradiation alone. In the functional layer, DS treatment during day-3 and 0 increased the expression of both adherens junction markers to 81% (β-catenin, *p* ≤ 0.001) and 83% (e-cadherin, *p* ≤ 0.001), respectively. From day 2 onwards, β-catenin and e-cadherin expression levels between 79% (day 2) and 88% (day 12 and day 14, maxima), with no significant difference to irradiation alone. The irradiation-induced increase of the staining intensity was not influenced by additional DS treatment for both adherens marker proteins.

#### 2.2.2. Tight Junctions—Claudin-1 and Occludin

In control specimen, 80% of germinal epithelial cells expressed claudin-1 and 81% expressed occludin. With the onset of fractionation, the percentage increased to roughly 95% for both proteins from day 4 onwards. In the functional compartment, 50% of cells expressed claudin-1 and 46% were found to be positive for occludin. During fractionation, claudin-1 expression increased to a maximum of 76% on day 10 (Figure 2i). Also occludin expression increased during irradiation treatment. A maximum of 83% was found on day 14 (Figure 2m). In the germinal layer, claudin-1 as well as occludin staining intensity increased from 1.4 a.u. on day 0 to 2.4 a.u. on day 14 (Figure 2j,n). In the functional tissue compartment, the staining intensity for both, claudin-1 and occludin, increased from 1.2 a.u. (day 0) to 2.7 a.u. (day 14) (Figure 2l,p).

Additional DS treatment led to an upregulation of both, claudin-1 and occludin in both tissue compartments already during the 3 days pretreatment. In the germinal epithelial layer, the only significant differences between radiation treatment alone and radiation plus DS were found on day 0 (claudin-1, *p* = 0.008 and occludin, *p* = 0.006).

In the functional epithelium, additional DS treatment significantly upregulated tight junction marker expression on every time point investigated. Both, claudin-1 and occludin were upregulated 15% (claudin-1, *p* = 0.001) and 20% (occludin, *p* ≤ 0.001) on day 0 due to the three days DS pretreatment compared to radiotherapy alone. Afterwards, DS-mediated claudin-1 upregulation was most pronounced on days 10 to 14. On day 10, 86% of functional epithelial cells were claudin-1 positive compared to 76% in mice receiving irradiation treatment only (*p* = 0.003), 90% vs. 74% on day 12 (*p* = 0.002) and 92% vs. 76% on day 14 (*p* = 0.0001). The highest occludin expression levels were found likewise on days 10 to 14 with 94% (IR + DS) vs. 76% (IR) on day 10 (*p* ≤ 0.001), 99% (IR + DS) vs. 78% (IR) on day 12 (*p* ≤ 0.001) and 98% vs. 83% on day 14 (*p* = 0.003).

In the germinal epithelial layer, DS increased the staining intensity of claudin-1 significantly on day 0 (*p* = 0.005), day 4 (*p* = 0.015) and day 12 (*p* = 0.043). No significant staining intensity differences were observed in the functional layer. Occludin staining intensity was significantly increased in the DS-treated experimental arm on day 0 (*p* = 0.022), day 4 (*p* = 0.008), day 12 (*p* = 0.023) and 14 (*p* = 0.0032) in the germinal layer and on days 4 (*p* = 0.008) and 10 (*p* = 0.032) in the functional tissue compartment.

### 2.3. Inflammation

#### 2.3.1. Epithelial NF-κB Expression

**Figure 3:**
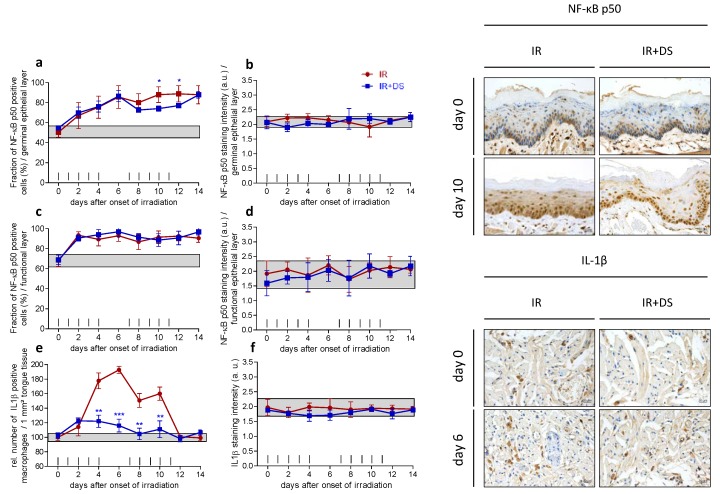
Effect of DS on irradiation-induced inflammatory changes. The relative number of NF-κB p50 positive epithelial cells and their respective staining intensity were analyzed in the germinal (**a**,**b**) and functional compartments of the lower tongue epithelium, respectively (**c**,**d**); IL-1β expression occurred exclusively in macrophages, which were scored in the deeper tongue tissue (**e**,**f**). Data points represent the mean of 5 animals, error bars indicate ±1 SD. The shaded areas illustrate the mean (±1 SD) from 5 control animals. The fractionation protocol is indicated on top of the abscissae. Histophotographs illustrate representative inflammatory marker staining on day 0 and after irradiation without and with DS without and with DS treatment on day 10 (NF-κB) and day 6 (IL-1β) of the study period. * *p* ≤ 0.05, ** *p* ≤ 0.01, *** *p* ≤ 0.001, Scale bar = 20 µm.

In control specimen, 50% of germinal and 68% of functional cells were positive for NF-κB p50. Expression increased during fractionation to a maximum of 89% in the germinal (Figure 3a) and to 93% in the functional epithelial layer on day 12 (Figure 3c). Additional DS administration reduced the NF-κB expression in the germinal layer on days 8 to 12. Significant differences were found on days 10 (*p* = 0.017) and days 12 (*p* = 0.015). No significant effect of DS treatment on the functional epithelial NF-κB expression was observed. The staining intensity stayed largely within control ranges throughout the study time in both epithelial compartments (Figure 3b,d).

#### 2.3.2. IL-1β Positive Macrophages

The number of IL-1β positive macrophages in control specimen was normalized to 100%. Within the first week of fractionation, the number of IL-1β positive macrophages peaked on day 6 with 193% of the control value. The relative number of IL-1β positive macrophages gradually decreased until normal values were restored on day 12. Additional DS application reduced the relative number of IL-1β positive macrophages significantly on day 4 (178% IR vs. 122% IR + DS, *p* = 0.003, day 6 (193% IR vs. 116% IR + DS, *p* ≤ 0.001), day 8 (151% IR vs. 105% IR + DS, *p* = 0.004) and day 10 (160% IR vs. 111% IR + DS, *p* = 0.008) (Figure 3e). The staining intensity of IL-1β remained within the control range as well as for irradiation with additional DS administration (Figure 3f).

### 2.4. Hypoxia

#### 2.4.1. HIF-1α

**Figure 4:**
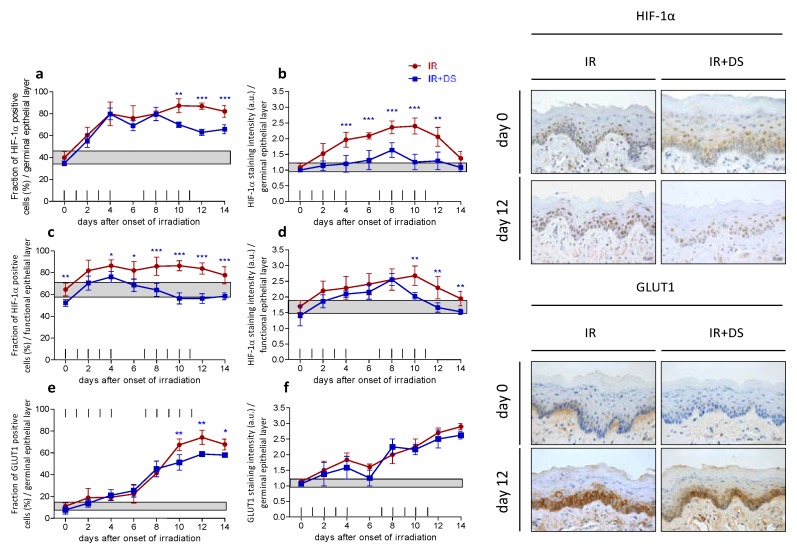
Effect of DS on irradiation-induced hypoxia. The relative number of HIF-1α positive epithelial cells and their respective staining intensity were analyzed in the germinal (**a**,**b**) and functional compartments of the lower tongue epithelium, respectively (**c**,**d**); GLUT1 expression was limited to the germinal tissue layer (**e**,**f**). Data points represent the mean of 5 animals, error bars indicate ±1 SD. The shaded areas illustrate the mean (±1 SD) from 5 control animals. The fractionation protocol is indicated on top of the abscissae. Histophotographs illustrate representative HIF-1α and GLUT1 staining on day 0 and after irradiation without and with DS treatment on day 12 of the study period. * *p* ≤ 0.05, ** *p* ≤ 0.01, *** *p* ≤ 0.001, Scale bar = 20 µm.

Fractionated irradiation stimulated a rapid expression increase of HIF-1α in both epithelial compartments from 40% in control specimen to a maximum of 87% on day 12 (germ. layer) and from 65% to 87% (funct. layer), on day 12 as well. Additional DS treatment reduced the expression significantly on day 10 (*p* = 0.002), day 12 (*p* ≤ 0.001) and day 14 (*p* = 0.001) in the germinal epithelium (Figure 4a). A significant DS-mediated reduction of HIF-1α in the functional layer was observed on day 10 (*p* ≤ 0.001) and day 12 (*p* ≤ 0.001) (Figure 4c). During fractionation, the HIF-1α staining intensity increased from 1 a.u. in control specimen to a maximum of 2.4 a.u. in the germinal layer and from 1.7 a.u. to 2.7 a.u. in the functional epithelium on day 8, respectively. Additional DS treatment reduced the HIF-1α staining intensity significantly in the germinal epithelium, on day 4 (*p* ≤ 0.001) day 6 (*p* ≤ 0.001), day 8 (*p* ≤ 0.001), day 10 (*p* ≤ 0.001) and day 12 (*p* = 0.002) (Figure 4b). In the functional epithelial layer, the HIF-1α staining intensity was reduced significantly after additional DS treatment on day 10 (*p* = 0.002), day 12 (*p* = 0.006) and day 14 (*p* = 0.005) (Figure 4d).

#### 2.4.2. GLUT1

GLUT1 expression was limited to the germinal layer. Fractionation increased the expression from 11% in control specimen to a maximum of 78% on day 12 (Figure 4e). The staining intensity increased from 1.1. a.u. in control samples to 2.9 a.u. on day 12 (Figure 4f). DS treatment reduced the relative number of GLUT1 positive epithelial cells significantly on day 10 (*p* = 0.005), day 12 (*p* = 0.005) and day 14 (*p* = 0.022) but did not influence the GLUT1 staining intensity throughout the treatment time.

## 3. Discussion

Oral mucositis is the most frequently occurring and dose-limiting early side effect of head-and-neck cancer radio(chemo)therapy. To date, no effective biology-based treatment was implemented in clinical routine [4]. Previously, we have described the radioprotective potential of dermatan sulfate (DS) in a preclinical model of radiotherapy-induced oral mucositis [12]. Systemic administration of 4 mg/kg DS resulted in a significant reduction of mucositis incidence, reduced mucositis duration and prolonged latent times until onset of ulcerations. Multiple administration schedules over varying time intervals have been tested with single dose as well as fractionated radiotherapy. The DS-associated mucoprotection was found to be most pronounced when DS was given over the longest time interval from day-3 before the onset of fractionation until the last day of two weeks of fractionated irradiation, i.e., day 11. This administration schedule was subsequently chosen for the present study, which was initiated to assess the DS-induced morphological and functional events underlying the observed radioprotective activity.

Fractionated irradiation rapidly and efficiently reduced epithelial proliferation, leading to subsequent mucosal hypoplasia, representing the primary cause of oral mucositis. Oral mucosa is a typical turnover tissue, characterized by proliferation in the germinal tissue compartments and continuous shedding of superficial cells. The equilibrium between proliferation and cell loss sustains a steady-state of the tissue. Radio(chemo)therapy interferes with proliferation in the germinal layer but, however, does not affect the differentiating cells in the functional layer and the rate of cell loss. Hence, ongoing cell shedding leads to epithelial hypoplasia and subsequently to mucosal lesions. Proliferative capacity recovered with the onset of the adaptive epithelial radiation response, repopulation [30], in the end of the first treatment week. Hence, cell numbers stabilized for the rest of the study period. This early phase of mucositis development was accompanied by inflammation and hypoxia, concordant to current literature [18,19,20,25,31,32]. We observed significant augmentation of epithelial conjunctions in the DS-treated experimental arm, indicating a DS-mediated reinforcement of epithelial integrity and cohesion. Likely, this presents the main parameter of the DS-mediated radioprotective effect in our preclinical model. Increased cellular conjunction presumably leads to a stronger anchorage within the epithelium, thus translating in increased cell numbers already prior to irradiation and during the treatment time without stimulating proliferation. Increased mucositis latency and shortened ulcer duration in the DS-treated experimental group, as reported previously [12], also indicate such a scenario. Likely, reduced irradiation-induced inflammation and DS-mitigated local hypoxia also contribute to these observations. However, while macrophage-associated IL-1β expression was systemically reduced, epithelial inflammation was marginally influenced with a significant reduction on two days during the second treatment week only. Likewise, the most pronounced reduction of epithelial hypoxia occurred in the second treatment week. Although the mucositis-mitigating effects of DS could partially be based on reduced inflammation and reduced local hypoxia, the main mechanism appears to be the reinforcement of epithelial cell cohesion based on the upregulation of tight as well as adherens junctions, likely via syndecan-1 (Sdc 1). Sdc 1 is rapidly released after injury, increases DS side chain expression and mediates reepithelialization during wound healing, with DS stimulating KGF function [33,34]. Sdc 1 was recently linked to intestinal mucosal barrier protection via enhancing tight junction protein expression [35]. We hypothesize similar mechanistic events in our model of oral mucositis, with exogenous DS stimulating Sdc 1 activity, which directly activates the expression of junction proteins. The reduced lesion duration we have observed in our previous study could also be based on the DS-mediated stimulation of Sdc 1 and KGF. Furthermore, a DS-mediated inhibition of P-selectin has been shown [36], which could facilitate both, reduced inflammation and ameliorated hypoxia. The latter could in addition be attributed to the DS-mediated activation of antithrombin 3, inhibiting factor Xa, and heparin co-factor 2, selectively inhibiting thrombin [37,38]. It appears that a combination of DS-associated activities advantageously interacted to mitigate radiotherapy-induced oral mucositis. It has been shown that inflammation and hypoxia increase epithelial permeability via reduction of epithelial junction proteins. Suppression of inflammation and hypoxia in turn protected junction complexes [39,40]. Similar synergies are highly likely for DS-mediated effects. Reduced hypoxia and mitigation of inflammation could additionally potentiate DS-mediated junctional protection. Importantly, the mucoprotective effects of DS are not based on stimulation of proliferation.

## 4. Material and Methods

All experiments were performed according to the current animal welfare legislation with approval by the respective authorities (file No. BMWF 66.009/0039-II/3b/2014, 04/02/2014).

### 4.1. Animals and Housing

For all experiments, mice of the inbred C3H/Neu strain from the breeding colony of the Department of Biomedical Research, Medical University Vienna, were used. Mice of both genders were included in the experiments. Mice were housed in a conventional environment with controlled temperature (22 ± 2 °C) and humidity (55 ± 10%) and a day/night rhythm of 12 h. The animals were housed in Makrolon^®^ cages, 1284L Eurostandard Type II L with a floor area of 530 cm^2^ (Techniplast GmbH, Hohenpeißenberg, Germany), maximum 5 animals per cage, on aspen wood bedding (ABEDD-LAB and VET Service GmbH, Vienna, Austria) and had free access to standard maintenance diet (ssniff Spezialdiäten GmbH, Soest, Germany) and fresh water ad libitum from standard Perspex drinking bottles. The age of the mice at the onset of the experiments ranged from 8 to 12 weeks.

### 4.2. Irradiation 

For all irradiation procedures, a YXLON Maxishot X-ray unit (Yxlon International X-ray GmbH, Hamburg, Germany) was used. Dosimetric commissioning was performed for the irradiation set-up. Standard dosimetric quality assurance was performed regularly and the dose-rate was found to be constant. Adjustment of the irradiation time thus defined the delivered dose. 

Fractionated irradiation with 3 Gy per day was given to the whole snouts of the animals. Un-anaesthetized animals were guided into a set-up of plastic tubes (inner diameter 2 cm). The snouts were positioned in conical holes (10 mm → 6 mm) of a Perspex block at the front end of the tubes. The rear ends were closed to prevent withdrawal of the animals. The bodies of the mice were shielded caudally from a plane from the eyes to the throat with 12 mm of the Pb-Bi-Sn alloy MCP-96. The treatment volume thus included the snouts with the entire tongue. The set-up for simultaneous irradiation of 8 animals was positioned in a standardized way in the central beam of the irradiation device. For fractionated irradiation, the YXLON Maxishot device was operated at 200 kV with a tube current of 20 mA and a focus size of 5.5 mm. A 4 mm Al and 0.6 mm Cu filter was used, which resulted in a dose rate of ca. 1 Gy/min at the focus-to-skin distance of 45.5 cm. The dose homogeneity between the individual snout positions was 3.2 ± 0.5%. The beam direction was vertical.

### 4.3. Experimental Protocol

**Figure 5:**
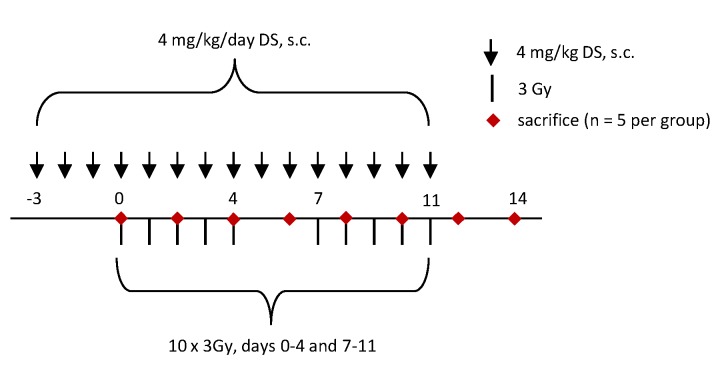
Experimental design. Daily fractionated irradiation was given in 10 fractions with 3 Gy per day over two weeks, including an irradiation-free weekend (days 5 and 6). Irradiation was either applied alone (group: IR) or in combination with daily 4 mg/kg dermatan sulfate (DS) treatment from day-3 before the start of irradiation (day 0) until sacrifice (group: IR + DS). 5 animals per experimental group were sacrificed in two days intervals.

The study comprised two experimental arms: irradiation alone (IR) and irradiation in combination with DS administration (IR + DS). DS (Sigma Aldrich, MO, USA, Cat No. C3788), dissolved in saline at a concentration of 1 mg/mL, was administered subcutaneously at a dose of 4 mg/kg from day-3 until the day before sacrifice; on irradiation days, the drug was given two hours after irradiation in the IR + DS arm. In both arms, groups of animals (*n* = 5) were sacrificed every second day, and their tongues excised at the base for further investigations. On the day of sacrifice, neither irradiation nor DS treatment was given. Five untreated and unirradiated mice served as a control group. The experimental protocol is illustrated in Figure 5.

### 4.4. Immunohistochemistry

Stainings were performed using the Leica Bond RX Automated Stainer (Leica Products/Equipment, Leica Microsystems, Inc., Buffalo Groove, IL, USA). The protocol used contained 30 min heating at 95 °C, dewaxing with the Leica Bond Dewax solution (Leica Biosystems, Inc., Buffalo Groove, IL, USA; Cat No. AR9222), antigen retrieval with the Bond Epitope Retrieval 1 solution (Leica Biosystems, Inc., Buffalo Groove, IL, USA; Cat No. AR9961) and blocking of unspecific binding sites with 2% goat serum. Primary antibody binding was visualized with diaminobenzidine chromogen and a hematoxylin counterstain, using the Leica Bond Refine Detection kit (Leica Biosystems, Inc., Buffalo Groove, IL, USA; Cat No. DS9800). Primary antibodies were diluted in the Leica Bond Antibody Diluent buffer (Leica Biosystems, Inc., Buffalo Groove, IL, USA; Cat No. AR9352) as follows: Anti-BrdU (Abcam, Cambridge, MA, USA; Cat No. 6671; rabbit polyclonal) 1:400, anti-Claudin-1 1:200 (Abcam, Cambridge, MA, USA; Cat No. 15098; rabbit polyclonal), anti-Occludin 1:200 (Abcam, Cambridge, MA, USA; Cat No. 222691; rabbit polyclonal), anti-E-cadherin 1:3000 (Abcam, Cambridge, MA, USA; Cat No. 76055; rabbit polyclonal), anti-β-catenin 1:700 (Abcam, Cambridge, MA, USA; Cat No. 2365; rabbit polyclonal), anti-IL-1β (Novus Biologicals, Littleton, CO, USA; Cat No. NBP1-19775; rabbit polyclonal) 1:700, anti-NF-κB p50 (Abcam, Cambridge, MA, USA; Cat No. 7971; rabbit polyclonal) 1:100, anti-GLUT-1 1:600 (Abcam, Cambridge, MA, USA; Cat No. 652; rabbit polyclonal) and anti-Hif-1α (Abcam, Cambridge, MA, USA; Cat No. 2185; rabbit polyclonal) 1:400.

### 4.5. Histological Analyses

Histological examination was performed with an Axio Lab.A1 HAL 35 (Carl Zeiss Microscopy, LLC, Thornwood, NY, USA) at a 400× magnification using an optical grid. The number of nucleated cells in the germinal and the functional (postmitotic) epithelial layer were counted per microscopic field (500 µm). Cellular morphology allowed a differentiation between germinal and functional tissue compartments. The total epithelial nucleated cell number was calculated as the sum of the germinal and functional values. Epithelial thickness was determined at two representative positions per section. Cell numbers and epithelial thickness of control specimen were set to 100%. Further values of irradiation-induced and/or DS-induced changes were calculated and are shown in relation to the control values. Epithelial cellularity, epithelial thickness and the fraction of cells positively stained for epithelial markers was determined in at least 5 microscopic fields, which corresponds to at least 2.5 mm epithelial length. BrdU incorporation, the expression of cell junction markers as well as hypoxia markers and NF-κB was assessed in the epithelium of the lower mouse tongue. The fraction of marker positive cells was evaluated separately for the germinal and the functional epithelial layers. IL-1β was not expressed by epithelial cells but in macrophages and hence was analyzed in the submucosal tongue tissue. Additionally, to the number of marker-positive cells, the respective staining intensity, corresponding to the amount of protein expressed, was assessed semiquantitatively with a score of arbitrary units (a.u.) from 0 (no signal), 1 (weak signal), 2 (intermediate signal) to 3 (strong signal). Epithelial morphology, marker-positive cells and their respective staining intensity were evaluated by 2 independent and experienced researchers in a blinded fashion after extensive training. Intra-observer variability was found to be negligible. Inter-observer variability was low and results in good agreement.

### 4.6. Statistical Analysis

For statistical analysis, the SPSS statistical software (IBM SPSS Statistics 21.0, SPSS Inc., Chicago, IL, USA) was used. Mean values and standard deviation (SD) were calculated for each animal, which then served to calculate the mean and standard error for each experimental group. The analysis of variance (one-way ANOVA) was used to test for the significance of a difference between the mean values. A *p*-value of ≤0.05 war regarded statistically significant. 

## 5. Conclusions

Dermatan sulfate has a significant mucositis-ameliorating potential. The radioprotective effect appears to be based mainly on an augmentation of epithelial junctions and thus strengthened epithelial integrity and reduced cell loss. No DS-associated stimulation of proliferation was observed. DS-mediated reduction of inflammation and local hypoxia may contribute to the mucoprotective activity. However, the impact of systemic DS treatment on the tumor radiation response needs to be assessed before DS may be considered in clinical studies.
